# Radiosynthesis and preclinical evaluation of [^68^Ga]Ga-NOTA-folate for PET imaging of folate receptor β-positive macrophages

**DOI:** 10.1038/s41598-020-70394-3

**Published:** 2020-08-12

**Authors:** Olli Moisio, Senthil Palani, Jenni Virta, Petri Elo, Heidi Liljenbäck, Tuula Tolvanen, Meeri Käkelä, Maxwell G. Miner, Erika Atencio Herre, Päivi Marjamäki, Tiit Örd, Merja Heinäniemi, Minna U. Kaikkonen, Fenghua Zhang, Madduri Srinivasarao, Juhani Knuuti, Philip S. Low, Antti Saraste, Xiang-Guo Li, Anne Roivainen

**Affiliations:** 1 Turku PET Centre, University of Turku, Kiinamyllynkatu 4-8, 20520 Turku, Finland; 2 Turku Center for Disease Modeling, University of Turku, Turku, Finland; 3 Turku PET Centre, Turku University Hospital, Turku, Finland; 4 A.I. Virtanen Institute for Molecular Sciences, University of Eastern Finland, Kuopio, Finland; 5 Institute of Biomedicine, University of Eastern Finland, Kuopio, Finland; 6 Department of Chemistry, Purdue University, West Lafayette, IN USA; 7 Heart Center, Turku University Hospital and University of Turku, Turku, Finland; 8 Turku PET Centre, Åbo Akademi University, Turku, Finland

**Keywords:** Translational research, Small molecules

## Abstract

Folate receptor β (FR-β), a marker expressed on macrophages, is a promising target for imaging of inflammation. Here, we report the radiosynthesis and preclinical evaluation of [^68^Ga]Ga-NOTA-folate (^68^Ga-FOL). After determining the affinity of ^68^Ga-FOL using cells expressing FR-β, we studied atherosclerotic mice with ^68^Ga-FOL and ^18^F-FDG PET/CT. In addition, we studied tracer distribution and co-localization with macrophages in aorta cryosections using autoradiography, histology, and immunostaining. The specificity of ^68^Ga-FOL was assessed in a blocking study with folate glucosamine. As a final step, human radiation doses were extrapolated from rat PET data. We were able to produce ^68^Ga-FOL with high radiochemical purity and moderate molar activity. Cell binding studies revealed that ^68^Ga-FOL had 5.1 nM affinity for FR-β. Myocardial uptake of ^68^Ga-FOL was 20-fold lower than that of ^18^F-FDG. Autoradiography and immunohistochemistry of the aorta revealed that ^68^Ga-FOL radioactivity co-localized with Mac-3–positive macrophage-rich atherosclerotic plaques. The plaque-to-healthy vessel wall ratio of ^68^Ga-FOL was significantly higher than that of ^18^F-FDG. Blocking studies verified that ^68^Ga-FOL was specific for FR. Based on estimations from rat data, the human effective dose was 0.0105 mSv/MBq. Together, these findings show that ^68^Ga-FOL represents a promising new FR-β–targeted tracer for imaging macrophage-associated inflammation.

## Introduction

Over-expression of folate receptor (FR) on cancer cells and during inflammatory responses has been used as a diagnostic and therapeutic tool to enable targeted delivery to tumors and sites of inflammation^1^. The beta isoform of the folate receptor (FR-β), distinctly expressed on activated macrophages, is a promising imaging marker for inflammatory conditions such as rheumatoid arthritis^2^. Currently, imaging of inflammation by positron emission tomography/computed tomography (PET/CT) is mainly performed using the glucose analog 2-deoxy-2-[^18^F]fluoro-*D*-glucose (^18^F-FDG), which reflects high consumption of glucose by macrophages and other inflammatory cell types. However, due to its non-specific nature, it is difficult to use ^18^F-FDG to detect inflammation adjacent to metabolically active tissues such as the heart. Therefore, the development of PET tracers targeting exclusive markers is vital for specific detection of inflammation. The FR-β–targeted PET tracers investigated for this purpose so far include [^18^F]AlF-NOTA-folate (^18^F-FOL)^3–5^, [^18^F]fluoro-PEG-folate^6^, and 3′-aza-2′-[^18^F]-fluoro-folic acid (^18^F-AzaFol)^7,8^; the latter two have already reached the initial clinical phase^9,10^. Other recently developed FR-targeted tracers include reduced ^18^F-folate conjugates^11^, ^55^Co-labeled albumin-binding folate derivatives^12^, and [^68^Ga]NOTA-folate^13^, all of which have been investigated in preclinical studies for imaging of FR-overexpressing tumors. Previously, we successfully used ^18^F-FOL PET to visualize FR-β–positive macrophages in mouse and rabbit models of atherosclerosis^3^. Atherosclerotic lesions exhibit chronic inflammation associated with accumulation of macrophages in the affected area, providing a rationale for investigating macrophage-targeted tracers.

**Figure 1:**
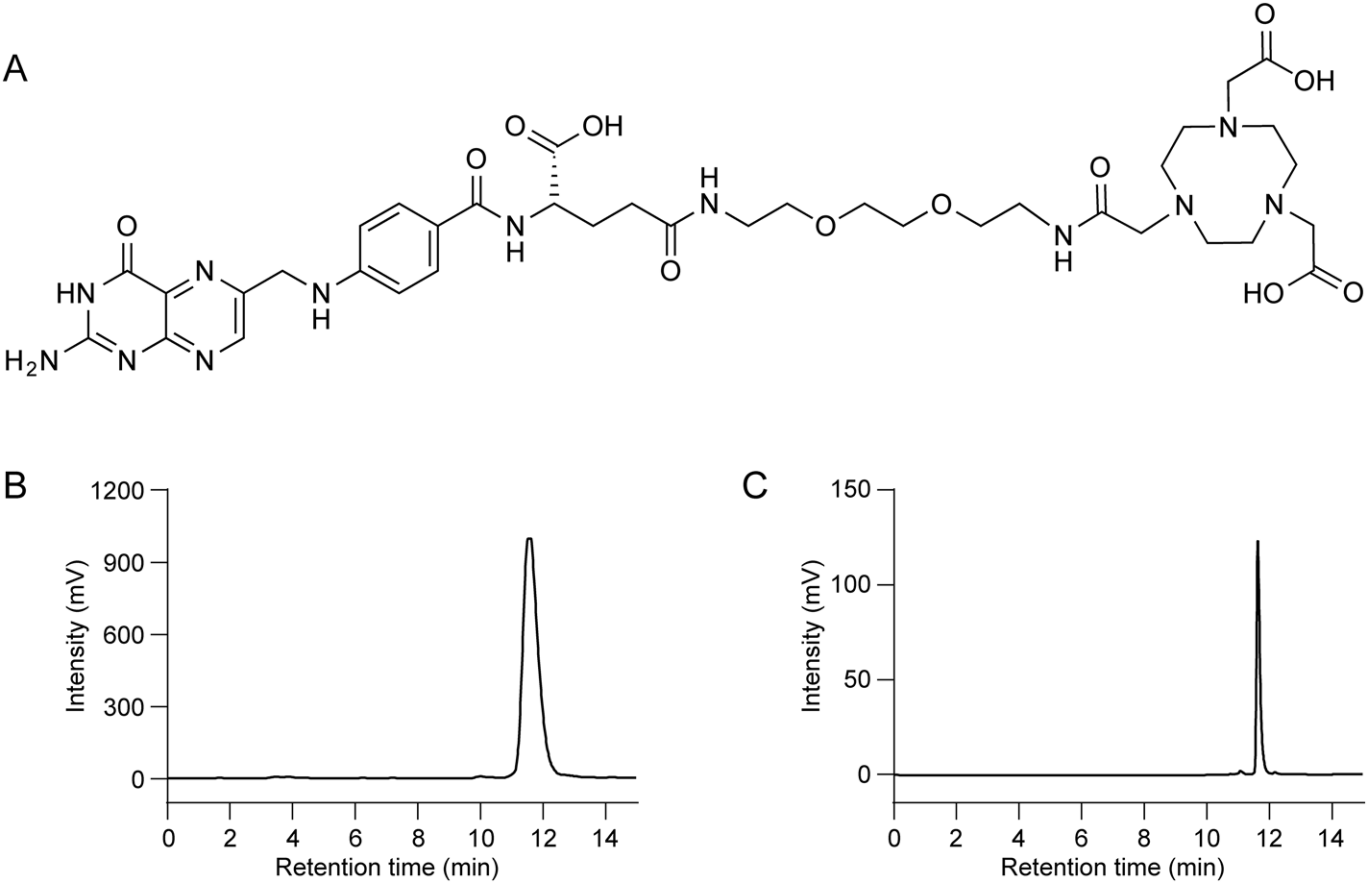
(**A**) The chemical structure of NOTA-Folate. The molecular weight of ^68^Ga**-**FOL is 908.82 Da. (**B**) Radio-HPLC chromatogram of ^68^Ga**-**FOL and (**C**) UV-chromatogram (280 nm) of 1 nmol NOTA-Folate precursor.

In radiosynthesis of ^68^Ga**-**radiopharmaceuticals, ^68^Ge/^68^Ga**-**generators are commonly used to obtain ^68^Ga**-**radionuclides; importantly, these generators can be conveniently implemented in a laboratory setting. In ^68^Ge/^68^Ga**-**generators, a certain amount of ^68^Ge is immobilized on a stationary phase, where the mother radionuclide decays into ^68^Ga, which can be eluted out for radiolabeling reactions. In this study, we prepared [^68^Ga]Ga-NOTA-folate (^68^Ga**-**FOL, Fig. 1) with generator-produced ^68^Ga and evaluated its potential for imaging of inflammation. ^68^Ga**-**FOL shares the same precursor structure as ^18^F-FOL^3^. Al^18^F labeling of NOTA-conjugates requires cyclotron facilities for the production of [^18^F]fluorine, whereas generator-produced ^68^Ga offers a convenient and cost-effective option for radiolabeling. First, we determined the binding affinity of ^68^Ga**-**FOL to human FR-β using transfected cells. Next, we investigated the uptake and specificity of intravenously (i.v.) administered ^68^Ga**-**FOL for the detection of inflamed atherosclerotic lesions in mice and compared the tracer with ^18^F-FDG. In addition, we determined the whole-body distribution kinetics in healthy rats, with and without the blocking agent folate glucosamine, and estimated the human radiation dose of ^68^Ga**-**FOL.

## Experimental section

### General materials and equipment

NOTA-folate precursor was synthesized as previously described^14^. ^68^GaCl_3_ was obtained from ^68^Ge/^68^Ga IGG-100 generators (Eckert & Ziegler, Valencia, CA, USA) by elution with 0.1 M hydrochloric acid (HCl) in water. TraceSELECT water (Honeywell, Morristown, NJ, USA) was used for radiosynthesis. Other chemicals were purchased from commercial suppliers. Chinese hamster ovary (CHO) cells stably transfected with human FR-β (CHO-hFRb; CHO-FR-β^+^) were a generous gift from Philip S. Low, Purdue University, USA. FR-β–negative CHO cells (CHO-FR-β^−^ control) were a generous gift from Sirpa Jalkanen, MediCity Research Laboratory, University of Turku, Finland. A dedicated small animal PET/CT (Inveon Multimodality; Siemens Medical Solutions, Knoxville, TN, USA) was used for PET/CT imaging, and a gamma counter (1480 Wizard 3″, PerkinElmer/Wallac, Turku, Finland or Triathler 3″, Hidex, Turku, Finland) was used for radioactivity measurement of ex vivo tissues, blood, and plasma samples. Tracer quality control and plasma metabolite analysis were performed using a LaChrom high-performance liquid chromatography (HPLC) system (Hitachi; Merck, Darmstadt, Germany) equipped with a Radiomatic 150TR flow-through radioisotope detector (Packard, Meriden, CT, USA) (radio-HPLC). Photomicroscopy images were taken with a digital slide scanner (Pannoramic 250 Flash or Pannoramic P1000; 3DHistec Ltd., Budapest, Hungary).

### ^68^Ga-FOL radiosynthesis

A fraction of ^68^Ga**-**eluate **(**0.5–1.0 mL) was mixed with an aqueous solution of 2-[4-(2-hydroxyethyl)piperazin-1-yl]ethanesulfonic acid (HEPES, 1.2 g/mL in 50–100 µL). NOTA-folate precursor (10–20 nmol in 20–40 µL water) was added, vortexed, and incubated for 10 min at 80 °C. The mixture was then cooled down and brought to a pH of ~ 6.5 by addition of 55 µL of 1 M sodium hydroxide (NaOH). The product was used without further purification. Radiochemical purity was analyzed primarily by HPLC. The HPLC conditions were as follows: 250 × 4.6 mm Jupiter Proteo 4µ C18 90 Å column (Phenomenex, Torrance, CA, USA); flow rate = 1 mL/min; wavelength λ = 220 nm; solvent A = 0.1% trifluoroacetic acid (TFA) in water; solvent B = 0.1% TFA in acetonitrile; gradient: during 0–14 min from 3% B to 35% B; during 14–15 min from 35% B to 3% B. Representative radio- and UV chromatograms are presented in Fig. 1B,C, respectively.

During radiosynthesis set-up, the radiochemical purity was also analyzed by instant thin-layer chromatography (iTLC) (Supplementary Figure S1). A 1.0 µL sample of the end product or reaction mixture was applied to a silica gel-based iTLC strip (iTLC-SG; Agilent, Santa Clara, CA, USA) and developed with 50 mM citric acid. Unbound ^68^Ga migrated up with the mobile phase with a retention factor (R_f_) of 0.8–1.0, while ^68^Ga**-**FOL remained at the application point (R_f_ = 0). To measure the unbound and tracer-bound ^68^Ga fractions, the strip was cut into two pieces along a line midway between the baseline and the solvent front, and each piece was measured separately in a gamma counter.

^68^Ge breakthrough was monitored by collecting aliquots from the ^68^Ge/^68^Ga generator eluate and measuring their radioactivities at time of collection and again 24 to 48 h later.

The lipophilicity of ^68^Ga**-**FOL (distribution coefficient Log*D*) was determined as previously described^15^. To evaluate ^68^Ga**-**FOL stability in the injectable formulation, we kept the end product at room temperature (RT) and took samples for radio-HPLC analysis at time intervals of up to 3 h.

### Quantification of Affinity of ^68^Ga-FOL for FR-β

The binding specificity of ^68^Ga**-**FOL to FR-β was evaluated using CHO-FR-β^+^ and CHO-FR-β^−^ cells (control). The cells were cultured at 37 °C in a CO_2_ incubator in RPMI 1640 medium (Gibco/Thermo Fisher Scientific, Waltham, MA, USA) supplemented with 10% fetal bovine serum (FBS; Biowest, Nuaillé, France).

To verify FR-β expression, the cultured cells were harvested and incubated with either fluorescein isothiocyanate (FITC)-conjugated anti-human FR-β antibody (m909^16^, a gift from Philip S. Low) or allophycocyanin (APC)-conjugated anti-human FR-β antibody (mouse IgG2a; BioLegend, San Diego, CA, USA) or the corresponding isotype controls (mouse IgG-FITC, mouse IgG2a-APC; BioLegend). The cells were fixed using paraformaldehyde and analyzed using a Fortessa fluorescence-activated cell sorting (FACS) device (BD Biosciences, Franklin Lakes, NJ, USA) and the Flowing software (Cell Imaging and Cytometry Core, Turku Bioscience, Turku, Finland).

After verifying the presence of FR-β on the cells, either CHO-FR-β^+^ or CHO-FR-β^−^ cells were cultured on one side of a 92 mm petri dish in a tilted position in growth medium at 37 °C in a CO_2_ incubator. The other side of the petri dish with no cells was used as background control for non-specific binding of ^68^Ga**-**FOL. Once the cells grew to a confluent monolayer, the growth medium from the petri dishes was removed, and phosphate-buffered saline (PBS) containing calcium and magnesium with 10% FBS (binding medium) was added. To starve the cells of folate, the dishes were incubated for 30 min at 37 °C in a CO_2_ incubator. After incubation, the cells were rinsed with binding medium (2 × 2 mL). A LigandTracer Yellow instrument (Ridgeview Instruments AB, Uppsala, Sweden) was used to measure the dissociation constant (K_*D*_) for ^68^Ga**-**FOL. The assay protocol with LigandTracer Yellow involves consecutive radioactivity measurements of the target (cell region) and the background (i.e., the cell-free region on the petri dish). Radioactivity was measured in each region for 30 s as raw counts per second (cps) with a delay of 5 s over the time course of the experiment. Target regions (cps) were corrected for background signal and radioactive decay. To detect background radioactivity or noise picked up by the instrument, 5 mL binding medium was added to the cells on the petri dish. After 15 min, ^68^Ga**-**FOL was added stepwise to achieve a concentration range of 1 to 80 nM, followed by replacement with fresh binding medium to measure the dissociation. The ratio of bound ^68^Ga**-**FOL (to the cells) to background (petri dish) and the K_*D*_ value were calculated using the TraceDrawer software (Ridgeview Instruments AB).

### Animal experiments

Low-density lipoprotein receptor-deficient mice expressing only apolipoprotein B100 (LDLR^−/−^ApoB^100/100^, strain #003000; Jackson Laboratory, Bar Harbor, ME, USA) were used to induce atherosclerosis. The mice were fed a high-fat diet (HFD; 0.2% total cholesterol, TD 88137, Envigo, Madison, WI, USA) starting at the age of 2 months and maintained for 3–5 months. C57BL/6JRj mice (Central Animal Laboratory of the University of Turku) fed with a regular chow diet were used as healthy controls. In total, 17 LDLR^–/–^ApoB^100/100^ (34.7 ± 5.5 g) and six healthy control mice (29.65 ± 1.9 g) were studied. In addition, six Sprague–Dawley rats (135.9 ± 17.1 g) from the Central Animal Laboratory of the University of Turku were studied.

All animals were housed at the Central Animal Laboratory of the University of Turku, and had ad libitum access to water and food throughout the study. All animal experiments were approved by the national Animal Experiment Board in Finland (license number ESAVI/4567/2018) and were carried out in compliance with European Union Directive 2010/63/EU.

### Mouse studies

#### PET/CT Imaging

The mice were fasted for 4 h prior to imaging, anesthetized with isoflurane (4–5% induction, 1–2% maintenance), and placed on a heating pad. The mice then received i.v. ^18^F-FDG (14.4 ± 0.2 MBq) via a tail vein cannula; the next day under the same conditions, they received ^68^Ga**-**FOL (20.1 ± 1.0 MBq). Immediately after PET, an iodinated contrast agent (100 µL eXIATM160XL; Binitio Biomedical, Ottawa, ON, Canada) was i.v. injected, and high-resolution CT was performed for anatomical reference. The Carimas 2.10 software (Turku PET Centre, Turku, Finland, www.turkupetcentre.fi/carimas/) was used to analyze PET/CT images. We defined regions of interest (ROIs) for the myocardium in coronal PET/CT images using the contrast-enhanced CT as an anatomical reference, as previously described^2^. The results were normalized against the injected radioactivity dose and animal body weight, i.e., the data were expressed as standardized uptake values (SUVs).

#### Ex vivo biodistribution

To study the specificity of ^68^Ga**-**FOL uptake, an in vivo blocking study was performed with another group of HFD-fed LDLR^–/–^ApoB^100/100^ mice i.v. injected with ^68^Ga**-**FOL alone or ^68^Ga**-**FOL in conjunction with a 100-fold molar excess of folate glucosamine. Mice were i.v. injected with ^68^Ga**-**FOL (11.3 ± 0.8 MBq) and euthanized after 60 min. Various tissues were excised and weighed, and their radioactivity was measured with a γ-counter (Triathler 3″, Hidex). After compensating for radioactivity remaining in the tail and cannula, the ex vivo biodistribution of ^68^Ga**-**radioactivity results were expressed as SUVs, and blocking and non-blocking results were compared.

#### Autoradiography, histology, and immunostainings

Following PET/CT imaging, the dissected aortic arch was processed into 20 and 8 µm cryosections. The 20 µm cryosections were used for digital autoradiography analysis as previously described^3^. Briefly, the sections were apposed on an Imaging Plate BAS-TR2025 (Fuji, Tokyo, Japan), and the plates were subsequently scanned on Fuji Analyzer BAS-5000 after an exposure time of 3 h for ^68^Ga**-**FOL and at least 4 h for ^18^F-FDG. After scanning, sections were stored at –70 °C until staining with hematoxylin–eosin (H&E) and then scanned with a Pannoramic digital slide scanner. Autoradiographs were analyzed using the Tina 2.1 software (Raytest Isotopemessgeräte, GmbH, Straubenhardt, Germany), and the uptake of ^68^Ga**-**FOL and ^18^F-FDG was corrected for injected radioactivity dose per unit body mass and radioactive decay during exposure; data were expressed as photostimulated luminescence per square millimeter (PSL/mm^2^). For immunohistochemistry, adjacent 8 µm sections were used to investigate co-localization of ^68^Ga**-**FOL with Mac-3-positive macrophages. The sections were incubated with anti-mouse Mac-3 antibody (1:1,000; BD Biosciences, Franklin Lakes, NJ, USA), and a color reaction was subsequently developed using 3.3′-diaminobenzidine (Bright-DAB, BS04-110).

#### In vivo stability

To determine the in vivo stability of ^68^Ga**-**FOL, plasma samples collected from atherosclerotic mice (*n* = 3) at 60 min post-injection were analyzed using radio-HPLC. Blood samples were collected in heparinized tubes and centrifuged at 4 °C for 5 min at 2,118 × *g*. Plasma proteins were precipitated with 10% sulfosalicylic acid (1:1 *v/v*), followed by centrifugation at RT for 2 min at 14,000×*g*. The supernatant was analyzed by radio-HPLC. Standard samples were prepared by addition of ^68^Ga**-**FOL tracer to 500 µL plasma supernatant collected from mice that had not received tracer. Both standard and metabolite samples applied to radio-HPLC analysis were normalized to a final volume of 1 mL by dilution with radio-HPLC solvent A if necessary. The radio-HPLC conditions were as follows: 250 × 10 mm Jupiter Proteo 5µ C18 90-Å column (Phenomenex); flow rate = 5 mL/min; solvent A = 0.1% TFA in water; solvent B = 0.1% TFA in acetonitrile; gradient, 0–11 min from 3% B to 25% B, 11–12 min from 25% B to 100% B, 12–14 min 100% B.

### Rat studies

To determine distribution kinetics and estimate human radiation dose, dynamic whole-body ^68^Ga**-**FOL PET/CT was performed in six healthy rats. In addition, three of the rats were also subjected to a blocking experiment with co-injection of a 100-fold excess of folate glucosamine. The rats were injected with 10.3 ± 0.4 MBq of ^68^Ga**-**FOL and PET imaged for 60 min. After imaging, the rats were euthanized, and various tissues were excised, weighed, and measured for radioactivity. Plasma samples were analyzed by radio-HPLC as described above. Using CT as an anatomical reference, quantitative PET image analysis was performed by defining ROIs on the main organs, and time–activity curves were extracted with the Carimas software. Human radiation dosimetry was estimated from the rat data using the OLINDA/EXM 2.2 software^17^.

### Statistical analysis

Results are presented as means ± SD. Differences between groups were analyzed by the unpaired Student *t *test using Microsoft Excel. *P *values < 0.05 were considered statistically significant.

## Results

### Radiosynthesis

^68^Ga**-**FOL was produced with a radioactivity concentration of 345.8 ± 118.9 MBq/mL (*n* = 13) and high radiochemical purity (96.6% ± 2.7%). Molar activity was 21.8 ± 6.9 GBq/µmol at the end of radiosynthesis. ^68^Ge-breakthough remained under 0.001% throughout the study. The distribution coefficient (Log *D*) of ^68^Ga**-**FOL was − 3.28 ± 0.33 (*n* = 3), indicating high hydrophilicity.

### In vitro quantification of ^68^Ga-FOL binding affinity to FR-β

**Figure 2:**
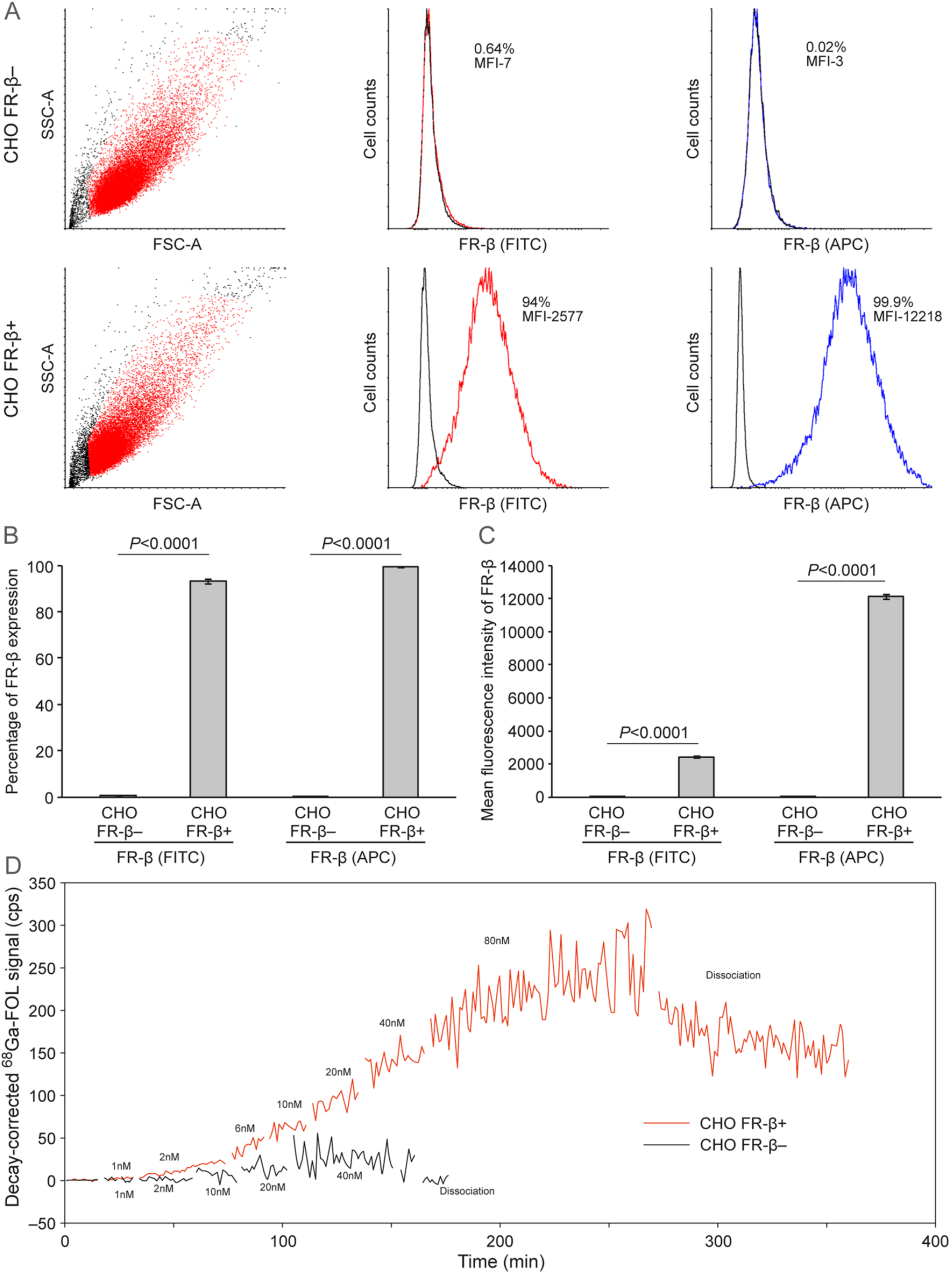
(**A**) Representative flow cytometry data showing FR-β expression on human FR-β–negative and FR-β–positive CHO cells stained with FITC-conjugated (red) or APC-conjugated anti–FR-β antibody (blue) or the corresponding isotope controls (black). Quantification of FR-β expression is presented as (**B**) percentage and (**C**) mean fluorescence intensity. (**D**) Representative real-time binding affinity of ^68^Ga**-**FOL, measured using LigandTracer. The graph generated with TraceDrawer shows raw counts per second (cps) after correction for background signal and radioactive decay.

FACS analyses confirmed that FR-β was clearly expressed on CHO-FR-β^+^ cells but not on CHO-FR-β^−^ cells (Fig. 2A–C). In binding assays, with a stepwise increase in the concentration of ^68^Ga**-**FOL from 1 to 80 nM, binding of ^68^Ga**-**FOL to CHO-FR-β^+^ cells was gradually increased and exhibited a K_*D*_ of 5.1 ± 1.1 nM (*n* = 3). By contrast, we observed no clear accumulation of ^68^Ga**-**FOL on CHO-FR-β^−^ cells, even at concentrations up to 40 nM (Fig. 2D).

### ^68^Ga-FOL detects macrophage-rich lesions in atherosclerotic mice

We evaluated the biodistribution of i.v.-administered ^68^Ga**-**FOL in mice using in vivo PET/CT, ex vivo gamma counting of excised tissues, and ex vivo autoradiography of aorta cryosections. To study the specificity of ^68^Ga**-**FOL to FR-β, we blocked by co-injecting a molar excess of folate glucosamine. In addition, we compared ^68^Ga**-**FOL with ^18^F-FDG in a head-to-head PET/CT imaging setting, as well as by ex vivo autoradiography.

The in vivo stability of ^68^Ga**-**FOL was high: 60 min after i.v. injection, the amount of intact tracer was 63.3% ± 1.2% of total plasma radioactivity in LDLR^–/–^ApoB^100/100^ mice (*n* = 3, Supplementary Figure S2).

Our ex vivo results revealed that the aortic uptake of ^68^Ga**-**FOL was higher in atherosclerotic mice (SUV 0.75 ± 0.12) than in healthy controls (SUV 0.41 ± 0.10, *P* = 0.004) or atherosclerotic mice from the blocking study (SUV 0.09 ± 0.03, *P* = 0.001). Furthermore, the concentration of radioactivity was threefold greater in atherosclerotic aorta than in blood (SUV 0.23 ± 0.09). The highest radioactivity uptake was in FR-positive kidneys^18^ in both atherosclerotic and control mice (SUV 22.30 ± 3.28 and 20.27 ± 5.48, respectively, *P* = 0.49), and uptake was significantly reduced in a blocking study performed in atherosclerotic mice (SUV 2.65 ± 1.80, *P* = 0.0002). The radioactivity of other tissues was much lower than that of the kidneys. Besides the kidneys, blocking with folate glucosamine in atherosclerotic mice decreased the radioactivity concentration in many other tissues as well, including an 88% reduction in the aorta (Supplementary Table S1).

**Figure 3:**
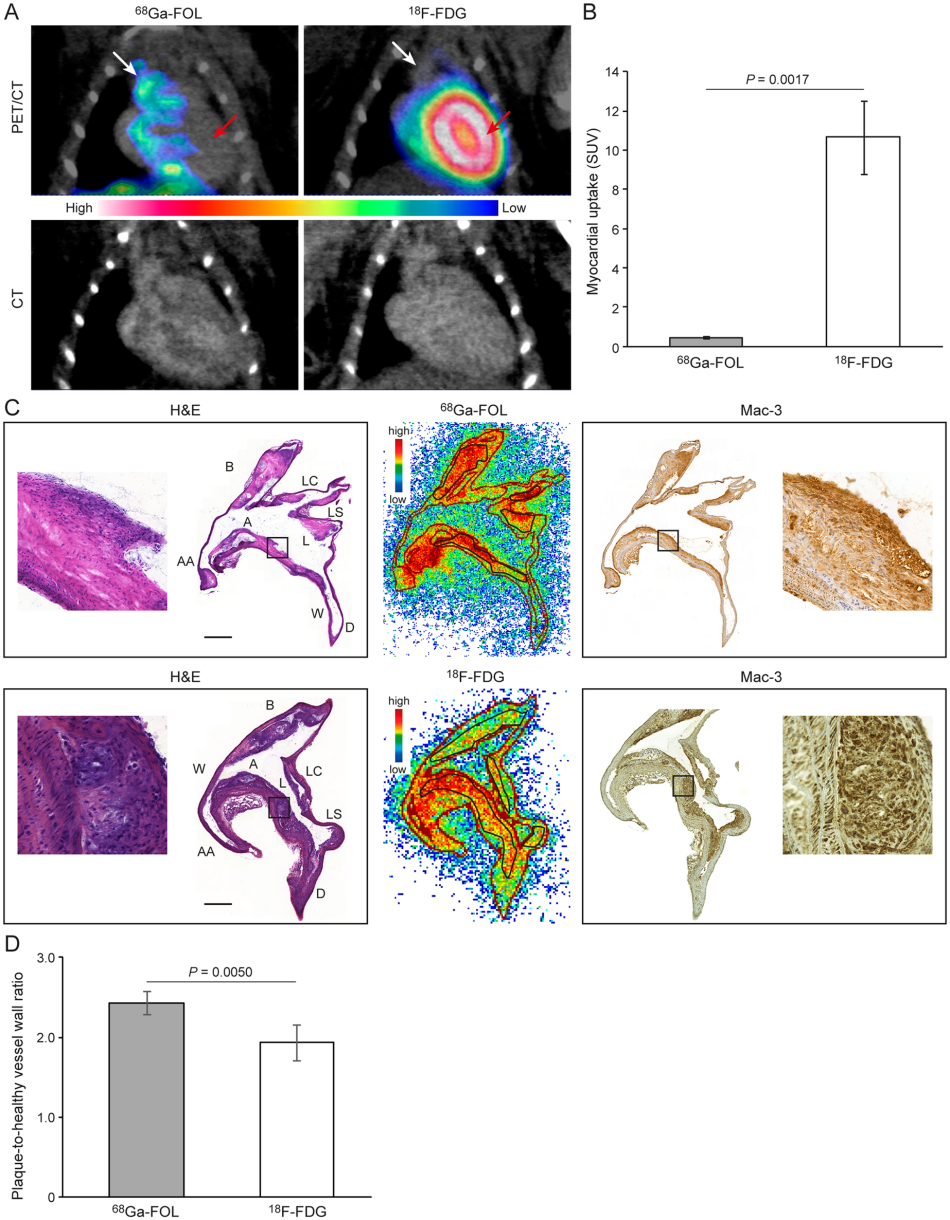
(**A**) Coronal PET/CT and CT images of an atherosclerotic mouse that was administered ^68^Ga**-**FOL or ^18^F-FDG. White arrows show the aortic arch, and red arrows show the myocardial region. (**B**) Quantification of myocardial PET data showing a significant difference between the tracers. (**C**) Hematoxylin–eosin (H&E) staining and autoradiography images from representative aorta cryosections, and Mac-3 macrophage marker staining in consecutive aorta cryosections. Black rectangles in the images indicate the plaque region, which are shown at higher magnification. Scale bar = 0.5 mm. A = arch; AA = ascending aorta; B = brachiocephalic artery; D = descending thoracic aorta; L = lesion; LC = left common carotid artery; LS = left subclavian artery; W = wall. (**D**) Quantification of autoradiography data showing a significant difference between the tracers.

A comparison of the two tracers by in vivo PET/CT revealed that myocardial uptake of ^68^Ga**-**FOL (SUV 0.43 ± 0.06) was significantly lower than that of ^18^F-FDG (SUV 10.6 ± 1.88, *P* = 0.001, Fig. 3A,B).

To further elucidate ^68^Ga**-**FOL and ^18^F-FDG uptake in the aortas of atherosclerotic mice in greater detail, we analyzed radioactivity using autoradiography and H&E staining of aortic cryosections followed by macrophage-detecting immunohistochemistry on adjacent tissue cryosections. The results revealed that ^68^Ga**-**FOL and ^18^F-FDG radioactivity co-localized with Mac-3–positive macrophage-rich plaques (Fig. 3C). The plaque-to-healthy vessel wall ratio of ^68^Ga**-**FOL (2.44 ± 0.15) was significantly higher than that of ^18^F-FDG (1.93 ± 0.22, *P* = 0.005, Fig. 3D).

### Distribution kinetics in rats and estimation of the human radiation dose of ^68^Ga-FOL

**Figure 4:**
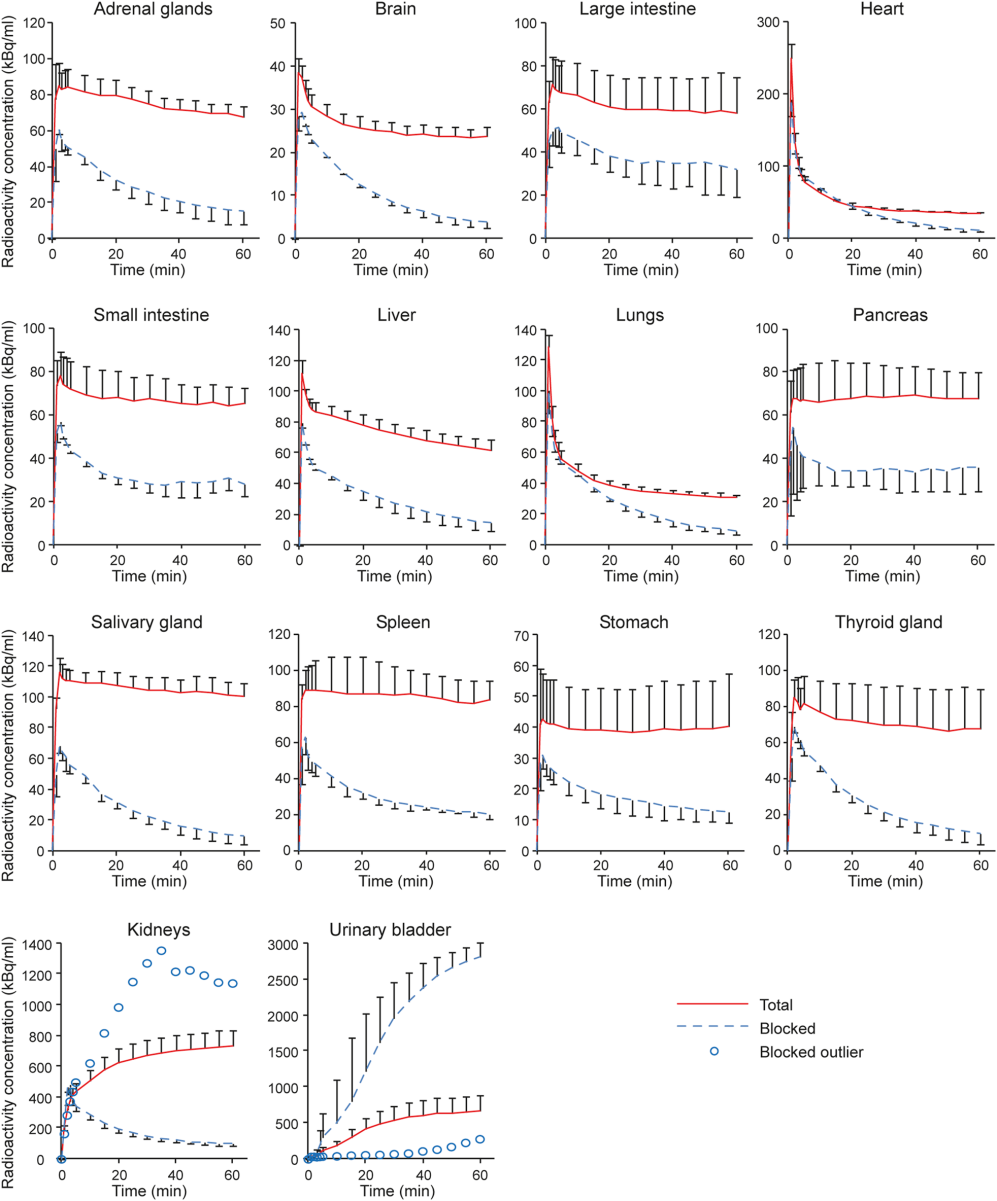
Time–activity curves of healthy rat tissues after i.v. injection of ^68^Ga**-**FOL, or co-injection of ^68^Ga**-**FOL and molar excess of folate glucosamine (blocking experiment).

In rats as in mice, ^68^Ga**-**FOL underwent fast renal excretion and highest uptake in kidneys, urine, salivary glands, liver, and spleen (Fig. 4). Co-injection of ^68^Ga**-**FOL along with a molar excess of folate glucosamine clearly decreased tracer uptake in several organs but increased urinary excretion. Radio-HPLC analysis of plasma samples revealed that ^68^Ga**-**FOL was relatively stable in vivo (Supplementary Figure S2); at 60 min post-injection, 71.8% ± 1.5% of the total radioactivity was from the intact tracer in healthy rats (*n* = 3) without blocking, and 88.0% ± 0.7% (*n* = 3, *P* = 0.0002) when blocked with folate glucosamine. Extrapolating from the rat PET data, the estimated human effective dose for a 73 kg man was 0.0105 mSv/MBq. The most critical organ was the kidney (0.1420 mSv/MBq) (Supplementary Table S2).

## Discussion

In this study, ^68^Ga**-**FOL was conveniently prepared with ^68^Ga**-**eluate from a ^68^Ge/^68^Ga**-**generator based on a fractionation elution method. We observed that ^68^Ga**-**FOL binds to FR-β with high affinity and accumulates in atherosclerotic lesions in mice following i.v. administration. Importantly, ^68^Ga**-**FOL exhibited lower myocardial uptake and higher plaque-to-healthy vessel wall ratio than ^18^F-FDG. Based on estimation from the rat data, the human radiation dose of ^68^Ga**-**FOL was low.

To produce ^68^Ga**-**FOL, we used a fractionation method to obtain ^68^GaCl_3_ from a ^68^Ge/^68^Ga**-**generator for a chelation reaction with the precursor compound NOTA-folate. This is a well-established method that we used previously, and the total radiosynthesis takes less than 20 min.

Previously, two folate-based imaging agents for single-photon emission computed tomography (SPECT), ^99m^Tc-EC20 and ^111^In-EC0800, were used to detect atherosclerotic lesions in mice^19,20^. Additionally, the PET tracer 3′-aza-2′-^18^F-fluorofolic acid can detect FR-β–positive macrophages in human atherosclerotic plaques in vitro^8^. In our previous studies, we demonstrated that ^18^F-FOL is specific for FR-β–positive macrophages and can detect inflamed atherosclerotic plaques in mice and rabbits, as well as in human tissue Sects. ^3^. However, the affinity of ^68^Ga**-**FOL binding to human FR-β has not been previously evaluated, and its ability to detect atherosclerotic lesions has not been compared with that of ^18^F-FDG. Our in vitro binding assay of ^68^Ga**-**FOL with CHO-FR-β^+^ and CHO-FR-β^−^ cells revealed specificity and high affinity for FR-β (5.1 ± 1.1 nM), close to the binding affinity of ^18^F-FOL (1.0 nM) for FR-positive tumor xenografts reported earlier^14^. Blocking studies in mice and rats further supported the tracer’s specificity for FR, and mouse studies confirmed the ability to detect macrophage-rich inflammatory lesions. The observed low myocardial uptake is beneficial for detection of atherosclerotic lesions in coronary arteries in prospective PET/CT studies of patients with coronary heart disease. When compared with our earlier ^18^F-FOL study, ^68^Ga**-**FOL exhibited similar plaque-to-healthy vessel wall ratios (2.44 ± 0.15 for ^68^Ga**-**FOL and 2.60 ± 0.58 for ^18^F-FOL). However, the in vivo stability of ^68^Ga**-**FOL in mice (63% ± 1% intact tracer at 60 min post-injection) was slightly lower than in our previous studies with ^18^F-FOL (85% ± 6% at 60 min post-injection)^3^. The human effective dose of ^68^Ga**-**FOL extrapolated from the rat data (0.0105 mSv/MBq) is low and within the same range as other ^68^Ga**-**tracers^21–23^.

We have obtained ^68^Ga**-**FOL with moderately high molar activity but the impact of molar activity on the imaging performance was not evaluated. On the other hand, we are also aware that high > 1,000 GBq/µmol molar activity does not always improve in vivo imaging, as reported by Wurzer and co-workers^24^. Our tracer, ^68^Ga**-**FOL, has directly conjugated NOTA, i.e. one carboxylic arm is used to form an amide bond, which makes the structure less optimal for ^68^Ga chelation than the N_3_O_3_ hexadentate coordination^25^. We did not explore if a higher molar activity could have been achieved by replacing the NOTA with for example a 1,4,7-triazacyclononane,1-glutaric acid-4,7-acetic acid (NODAGA)^26^ or triazacyclononane-phosphinate (TRAP)^27^ chelator, which conserve the N_3_O_3_ coordination for ^68^Ga binding. This is our first-generation conjugate and further optimizations with better chelators such as NODAGA, or even more recent ones such as tris(hydroxypyridinone) (THP) and desferrioxamine (DFO)^28^ are indeed warranted. In this particular lead compound, NOTA was chosen since it is a commonly used chelator for radiolabeling with both [^18^F]AlF and ^68^Ga. Although NOTA is not the most optimized chelator for ^68^Ga, it is indeed applicable in the preparation of ^68^Ga**-**radiopharmaceuticals for clinical use^29^.

## Conclusions

In summary, we have prepared ^68^Ga**-**FOL and evaluated its FR-β targeting and imaging performance in vitro and in vivo. The preclinical results of ^68^Ga**-**FOL are in line with those of our previous studies using ^18^F-FOL and corroborate FR-β as an imaging target for detection of inflamed atherosclerotic lesions.

## Supplementary information

Supplementary information.
