# Supplementary material for: Radiosynthesis and preclinical evaluation of [68Ga]Ga-NOTA-folate for PET imaging of folate receptor β-positive macrophages
Source: Sci Rep. 2020 Aug 12;10:13593. doi: 10.1038/s41598-020-70394-3 (PMC7423886; doi:10.1038/s41598-020-70394-3)
Supplement: Supplementary file 1 — Supplementary information. [file 41598_2020_70394_MOESM1_ESM.pdf]

Supplementary Information for:

Radiosynthesis and Preclinical Evaluation of [^{68}Ga]Ga-NOTA-Folate for PET Imaging of Folate Receptor β-Positive Macrophages

Olli Moisio[1#], Senthil Palani[1#], Jenni Virta[1], Petri Elo[1], Heidi Liljenbäck[1,2], Tuula Tolvanen[3], Meeri Käkelä[1], Maxwell G. Miner[1], Erika Atencio Herre[1], Päivi Marjamäki[1], Tiit Örd[4], Merja Heinäniemi[5], Minna U. Kaikkonen[4], Fenghua Zhang[6], Madduri Srinivasarao[6], Juhani Knuuti[1,3], Philip S. Low[6], Antti Saraste[1,3,7], Xiang-Guo Li[1,8], and Anne Roivainen[1,2,3*]

[1] *Turku PET Centre, University of Turku, Turku, Finland*

[2] *Turku Center for Disease Modeling, University of Turku, Turku, Finland*

[3] *Turku PET Centre, Turku University Hospital, Turku, Finland*

[4] *A.I. Virtanen Institute for Molecular Sciences, University of Eastern Finland, Kuopio, Finland*

[5] *Institute of Biomedicine, University of Eastern Finland, Kuopio, Finland*

[6] *Department of Chemistry, Purdue University, West Lafayette, Indiana, United States of America*

[7] *Heart Center, Turku University Hospital and University of Turku, Turku, Finland*

[8] *Turku PET Centre, Åbo Akademi University, Turku, Finland*

#Equal contribution

*Correspondence: Anne Roivainen, Turku PET Centre, Kiinamyllynkatu 4-8, FI-20520 Turku, Finland. Tel: +35823132862, E-mail: anne.roivainen@utu.fi.

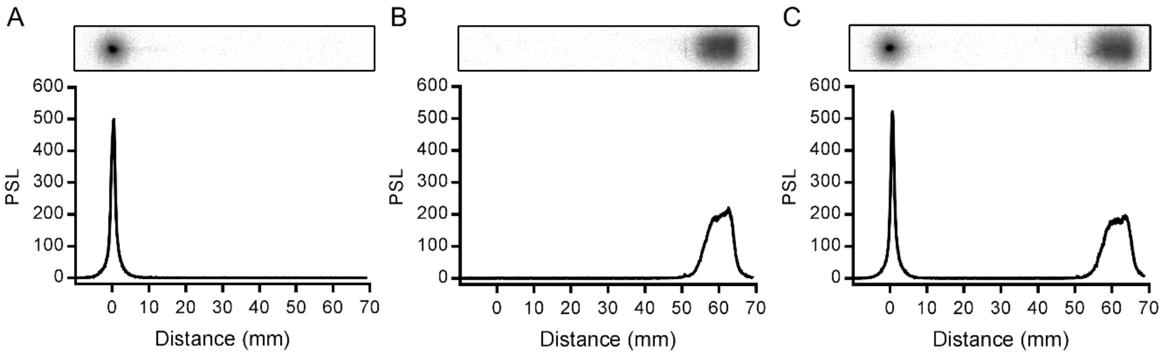

Figure S1. Representative autoradiographs (upper panels) and chromatograms (lower panels) of instant thin-layer silica gel (iTLC-SG) strips developed with 50 mM citric acid for (A) ^{68}Ga-FOL, (B) unbound ^{68}Ga in the reaction mixture without NOTA-folate precursor, and (C) co-application of ^{68}Ga-FOL and ^{68}Ga.

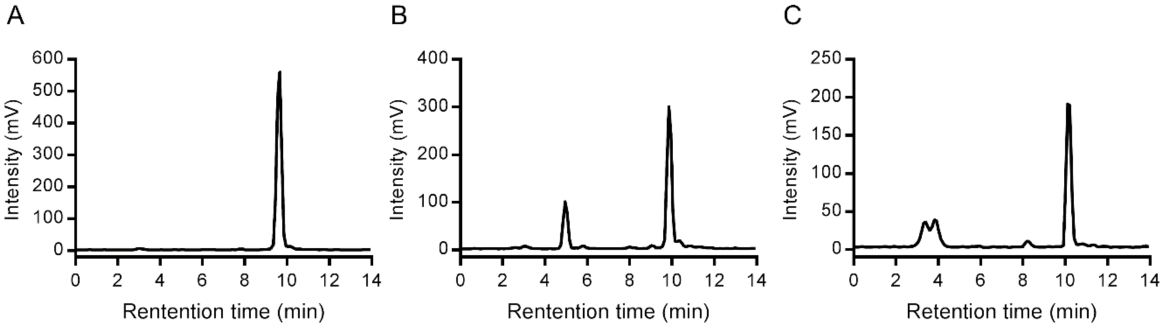

Figure S2. Representative radio-HPLC chromatograms of (A) ^{68}Ga-FOL standard, (B) rat plasma 60 minutes after ^{68}Ga-FOL injection, and (C) mouse plasma 60 minutes after ^{68}Ga-FOL injection.

Table S1. *Ex vivo* biodistribution of ^{68}Ga-FOL 60 minutes post-injection in mice, expressed as standardized uptake values (means ± SD)

**Tissue**	LDLR$^{-/-}$ApoB$^{100/100}$ atherosclerotic mice (*n* = 4)	LDLR$^{-/-}$ApoB$^{100/100}$ atherosclerotic mice, blocking (*n* = 4)	C67BL/6JRj control mice (*n* = 6)
**Aorta**	0.75 ± 0.12	0.09 ± 0.03 (*P* = 0.001)[a]	0.41 ± 0.10 (*P* = 0.004)[b]
**Brown adipose tissue**	0.29 ± 0.04	0.06 ± 0.02 (*P* = 0.006)[a]	0.31 ± 0.05 (*P* = 0.51)[b]
**Blood**	0.23 ± 0.09	0.29 ± 0.10 (*P* = 0.48)[a]	0.25 ± 0.14 (*P* = 0.84)[b]
**Bone (skull)**	0.13 ± 0.05	0.06 ± 0.02 (*P* = 0.04)[a]	0.16 ± 0.04 (*P* = 0.42)[b]
**Bone + marrow (femur)**	0.24 ± 0.18	0.07 ± 0.01 (*P* = 0.17)[a]	0.18 ± 0.04 (*P* = 0.57)[b]
**Brain**	0.08 ± 0.01	0.02 ± 0.00 (*P* = 0.001)[a]	0.06 ± 0.01 (*P* = 0.02)[b]
**Heart**	0.20 ± 0.02	0.05 ± 0.02 (*P*<0.0001)[a]	0.24 ± 0.03 (*P* = 0.04)[b]
**Intestine, small (empty)**	0.44 ± 0.06	0.42 ± 0.10 (*P* = 0.84)[a]	0.47 ± 0.27 (*P* = 0.76)[b]
**Intestine, large (empty)**	0.73 ± 0.14	0.19 ± 0.06 (*P* = 0.003)[a]	0.66 ± 0.08 (*P* = 0.38)[b]
**Kidneys**	22.30 ± 3.28	2.65 ± 1.80 (*P* = 0.0002)[a]	20.27 ± 5.48 (*P* = 0.49)[b]
**Lungs**	0.46 ± 0.03	0.19 ± 0.04 (*P* < 0.0001)[a]	0.39 ± 0.06 (*P* = 0.05)[b]
**Liver**	1.04 ± 0.43	0.33 ± 0.13 (*P* = 0.04)[a]	0.76 ± 0.16 (*P* = 0.29)[b]
**Lymph node**	1.37 ± 0.57	0.14 ± 0.02 (*P* = 0.02)[a]	4.07 ± 0.73 (*P* = 0.0002)[b]
**Muscle**	0.20 ± 0.02	0.05 ± 0.01 (*P* < 0.0001)[a]	0.13 ± 0.02 (*P* = 0.0003)[b]
**Pancreas**	0.43 ± 0.06	0.07 ± 0.02 (*P* = 0.0007)[a]	0.37 ± 0.03 (*P* = 0.17)[b]
**Plasma**	0.40 ± 0.15	0.48 ± 0.13 (*P* = 0.44)[a]	0.34 ± 0.15 (*P* = 0.61)[b]
**Spleen**	0.23 ± 0.07	0.15 ± 0.05 (*P* = 0.11)[a]	0.28 ± 0.13 (*P* = 0.42)[b]
**Stomach (empty)**	0.78 ± 0.14	0.25 ± 0.05 (*P* = 0.002)[a]	0.65 ± 0.12 (*P* = 0.17)[b]
**Thymus**	0.42 ± 0.13	0.06 ± 0.01 (*P* = 0.01)[a]	0.33 ± 0.08 (*P* = 0.25)[b]
**White adipose tissue**	0.22 ± 0.16	0.03 ± 0.01 (*P* = 0.10)[a]	0.30 ± 0.08 (*P* = 0.40)[b]

The blocking study was performed by injecting a 100-fold molar excess of folate glucosamine 1 minute before ^{68}Ga-FOL.
[a] Difference between LDLR$^{-/-}$ApoB$^{100/100}$ atherosclerotic mice *vs.* atherosclerotic mice + blocking, as assessed by independent-samples *t*-test.
[b] Difference between LDLR$^{-/-}$ApoB$^{100/100}$ atherosclerotic *vs.* C57BL//6JRj control mice, as assessed by independent-samples *t*-test.

Table S2. Human dose equivalent estimates (mSv/MBq) extrapolated from rat PET data

Organ	Organ doses
Adrenals	0.0243
Brain	0.0043
Esophagus	0.0121
Eyes	0.0110
Gallbladder wall	0.0139
Left colon	0.0140
Small intestine	0.0180
Stomach wall	0.0131
Right colon	0.0184
Rectum	0.0134
Heart wall	0.0078
Kidneys	0.1420
Liver	0.0118
Lungs	0.0069
Pancreas	0.0135
Prostate	0.0134
Salivary glands	0.0119
Red marrow	0.0102
Osteogenic cells	0.0096
Spleen	0.0173
Testes	0.0119
Thymus	0.0120
Thyroid	0.0113
Urinary bladder wall	0.0132
Total body	0.0130
Effective dose	0.0105
